# Detecting outliers beyond tolerance limits derived from statistical process control in patient‐specific quality assurance

**DOI:** 10.1002/acm2.14154

**Published:** 2023-09-08

**Authors:** Hong Qi Tan, Kah Seng Lew, Yun Ming Wong, Wen Chuan Chong, Calvin Wei Yang Koh, Clifford Ghee Ann Chua, Ping Lin Yeap, Khong Wei Ang, James Cheow Lei Lee, Sung Yong Park

**Affiliations:** ^1^ Division of Radiation Oncology National Cancer Centre Singapore Singapore Singapore; ^2^ Division of Physics and Applied Physics Nanyang Technological University Singapore Singapore; ^3^ Oncology Academic Clinical Programme Duke‐NUS Medical School Singapore Singapore

**Keywords:** outlier detection, patient‐specific quality assurance, portal dosimetry, statistical process control

## Abstract

**Background:**

Tolerance limit is defined on pre‐treatment patient specific quality assurance results to identify “out of the norm” dose discrepancy in plan. An out‐of‐tolerance plan during measurement can often cause treatment delays especially if replanning is required. In this study, we aim to develop an outlier detection model to identify out‐of‐tolerance plan early during treatment planning phase to mitigate the above‐mentioned risks.

**Methods:**

Patient‐specific quality assurance results with portal dosimetry for stereotactic body radiotherapy measured between January 2020 and December 2021 were used in this study. Data were divided into thorax and pelvis sites and gamma passing rates were recorded using 2%/2 mm, 2%/1 mm, and 1%/1 mm gamma criteria. Statistical process control method was used to determine six different site and criterion‐specific tolerance and action limits. Using only the inliers identified with our determined tolerance limits, we trained three different outlier detection models using the plan complexity metrics extracted from each treatment field—robust covariance, isolation forest, and one class support vector machine. The hyperparameters were optimized using the F1‐score calculated from both the inliers and validation outliers’ data.

**Results:**

308 pelvis and 200 thorax fields were used in this study. The tolerance (action) limits for 2%/2 mm, 2%/1 mm, and 1%/1 mm gamma criteria in the pelvis site are 99.1% (98.1%), 95.8% (91.1%), and 91.7% (86.1%), respectively. The tolerance (action) limits in the thorax site are 99.0% (98.7%), 97.0% (96.2%), and 91.5% (87.2%). One class support vector machine performs the best among all the algorithms. The best performing model in the thorax (pelvis) site achieves a precision of 0.56 (0.54), recall of 1.0 (1.0), and F1‐score of 0.72 (0.70) when using the 2%/2 mm (2%/1 mm) criterion.

**Conclusion:**

The model will help the planner to identify an out‐of‐tolerance plan early so that they can refine the plan further during the planning stage without risking late discovery during measurement.

## 1 INTRODUCTION

Pre‐treatment patient‐specific quality assurance (PSQA) is an integral part of any radiotherapy workflow to ensure the actual dose received by the patients are similar to the planned dose. By doing so, it serves to detect errors arising from beam modeling deficiency,^1^, ^2^ LINAC output change,^3^ or complex intensity modulated radiotherapy (IMRT) or volumetric arc radiotherapy (VMAT) treatment plans.^4^, ^5^ Unlike daily or monthly machine QA tolerances, which are laid out clearly in TG142,^6^ the method of PSQA and its action limit varies between hospitals.^7^, ^8^ TG218^9^ aims to fill this gap by offering recommendations and guidelines for PSQA practice. Following the recommendation, action and tolerance limit are defined on the gamma passing rate (GPR)^10^ result to identify an unacceptable treatment plan. According to TG218, action limit is defined as the permissible deviation in the PSQA results without risking harm to the patient, and tolerance limit is the boundary within which a process can be considered to be operating normally. Measurement results violating tolerance limit should be investigated before it transgresses the action limit. Either way, in the event that the tolerance or action limit is violated, a series of troubleshooting steps will ensue and replanning will be the final resort to reduce the plan complexity. This often leads to treatment delays and even stressful situations between different professions in the department. As such, the ability to predict whether a plan will violate the PSQA tolerance limit during planning phase is desirable to mitigate the above‐mentioned risk.

The most common approach to build a PSQA prediction model is based on plan complexity metrics extracted from a plan. A complex plan^11^, ^12^ has an increased sensitivity to machine delivery deviation and patient geometry variation, and poorer dose calculation accuracy through the multileaf collimator (MLC).^5^ The metrics can be further stratified into fluence, delivery and accuracy metrics depending on the approaches to calculate them.^13^ A recent review paper by A. Osman et al^14^ have shown that many groups have achieved a good prediction score for GPR by applying machine learning techniques with the metrics. Most of the studies treat the prediction of GPR as a regression task (predicting the numerical value of GPR) by using poisson regression,^15^, ^16^, ^17^ XGBoost,^18^, ^19^ random forest,^18^, ^19^, ^20^ and ANN^20^, ^21^ models. It is also possible to treat this as a classification problem to predict the passing or failing of the plan directly. However, a GPR threshold will have to be selected before training the classification model, whereas regression modelling allows a greater flexibility of deciding the threshold later after predicting the numerical value of GPR. Deep learning approach^22^, ^23^ is also becoming increasingly popular due to a greater degree of freedom for the model to explore the relationship between the plan and the PSQA result.

In this work, we will be using the plan complexity metrics to predict our local PSQA results measured using portal dosimetry (PD), for plans originating from two different treatment sites—pelvis and thorax. Rather than using the 3%/2 mm gamma criterion as stated in the TG218, we calculated the PSQA results using a more sensitive gamma criterion of 2%/2 mm, 2%/1 mm, and 1%/1 mm for this study.^24^, ^25^ As a result of this, we have to derive our institutional tolerance and action limits for our PD PSQA process. We will be using statistical process control (SPC)^26^, ^27^ methods to derive the site and criterion‐specific tolerance and action limits. SPC methods have been used widely in radiotherapy to monitor various QA processes^26^, ^28^, ^29^, ^30^, ^31^, ^32^ to make sure the process is in control and any deviation beyond random fluctuation can be identified promptly. The reason for having a site‐specific limit is that the nature of the plans are different between the two sites due to different organ‐at‐risk (OARs) and different MLC modulation required.^33^, ^34^

Contrary to other related works which developed prediction model to predict the actual GPR value (regression problem) or the event of GPR exceeding a certain defined threshold (classification problem), we developed an outlier detection model to predict the violation of the tolerance limit. This is because using the tolerance limit defined by the SPC procedure, the number of outliers (or violation of tolerance limits) are around 2–5%, which is challenging to model with a classification machine learning algorithm due to extreme class imbalance.^35^, ^36^ Hence, we will instead, develop an outlier detection model using all the inliers in this study and their corresponding plan complexity features, and finally evaluate the performance of our model on a validation dataset comprising of the outliers and inliers. Outlier detection differs from classification problem in two aspects.^36^, ^37^ First, while classification algorithm is trained on labelled data with two or more distinct classes, unsupervised outlier detection trains the model on a *single* class or unlabeled class where all the data are verified and regarded as inliers or normal observations; outliers are identified and excluded from the model training process. Second, the resulting decision boundaries are different between the two algorithms. In classification algorithm, the decision boundary will focus on partitioning the feature space to have correct class predictions. On the other hand, the decision boundary in outlier detection algorithm can be seen as a minimal volume hypersurface to enclose all the training data or inliers. It is widely used in detecting financial fraud, intrusion or detecting malfunctioning equipment.^37^ Outlier detection model does not necessarily yields a better performing model than traditional classification model. The main advantage is the ease of training a discriminatory model in a large dataset with extreme class imbalance. This is the first time outlier detection model is used in the prediction of PSQA result.

## 2 METHODS

### 2.1 Patient‐specific quality assurance data and methodology

The PSQA results from stereotactic body radiotherapy (SBRT) treatment from January 2020 to December 2021 are used in this study. SBRT is chosen due to the centre's practice of performing only PSQA measurement for hypofractionated treatment. The inclusion criteria for final data selection are as follows: 1) thorax and prostate SBRT, 2) VMAT, 3) 10FFF beam, and 4) TrueBeam (Varian Medical System, Palo Alto, California, USA) LINAC with Millennium 120 MLC. There are no specific exclusion in this study. With these criteria, we analyzed a total of 308 and 200 pelvis and thorax treatment fields, respectively. Of which, 211 pelvis and 138 thorax fields are measured in 2021. A treatment field is defined as a VMAT arc (full or partial) in this manuscript. All the field sizes in the PSQA are greater than 3 cm even for lung SBRT and thus does not fall under the small field regime.^38^ The PSQA is conducted using portal dosimetry (PD) with a source imager distance (SID) of 140 cm. Imager calibration is always performed prior to daily PD PSQA to ensure proper pixel correction to account for any radiation damage. The resulting PD measurement is compared with the planned dose using gamma analysis tool in Eclipse v13.6 (Varian Medical System, Palo Alto, California, USA). A low dose threshold of 10% is set and comparison is conducted using global percentages for the doses. The gamma passing rates (GPRs) are then recorded for each field using the 2%/2 mm, 2%/1 mm, and 1%/1 mm criteria. This is the perpendicular field by field (PFF) measurement method as defined in TG218.

### 2.2 Plan complexity metrics

Plan complexity metrics are extracted for all the plans in Eclipse treatment planning system (TPS) using an in‐house script developed with Eclipse scripting API. A total of 17 metrics are extracted from the plan and the acronyms together with their corresponding full names are shown in Table 1. Apart from TGi which is defined by W. Que et al.,^39^ the rest of the metrics are part of the deliverability and accuracy metrics as defined by S. Chiavassa et al.^11^ Feature 1, 8, 9, 12, 16, and 17 constitutes the deliverability metrics, which quantify the ability of the plan to meet the required mechanical motion and dosimetric dose rate. The rest of the metrics are part of the accuracy metrics which quantify the ability of the TPS in modeling the x‐ray fluence through the MLC accurately. In theory, a maximal set of plan complexity metrics should be extracted and used for predictive modeling to identify the best discriminating model. However, we choose only 17 metrics mainly due to the availability of the required parameters through the API.

**TABLE 1:** List of plan complexity metrics extracted from the plan and are used in the outlier detection model.

No.	Acronym	Actual name	No.	Acronym	Actual name
1	MCS	Modulation complexity score	9	MU_cp	MU per control point
2	mlcwidth	Average MLC width	10	CoA	Circumference over area
3	Fs	Average field size	11	MFA	Mean field area
4	SAS_5 mm	Small aperture score for aperture < 5 mm	12	AI	Aperture irregularity
5	SAS_10 mm	Small aperture score for aperture < 10 mm	13	EM	Edge metrics
6	SAS_20 mm	Small aperture score for aperture < 20 mm	14	MAD	Mean assymetric distance
7	TGi	Tongue and groove index	15	CAS	Cross axis score
8	MU_Gy	MU per Gray	16	AAV	Aperture area variability
			17	LSV	Leaf sequence variability

### 2.3 Statistical process control for determining tolerance and action limit

Statistical process control uses statistical method to monitor and control a process, which in this context, is the PSQA measurement. Control charts together with the statistically determined tolerance and action limits help to ensure the process is under control and any deviation can be detected promptly. In this work, the datasets are divided into thorax and pelvis sites to calculate site‐specific tolerance and action limits.

### 2.4 The action limit is determined using

(1) $$\mathrm{AL}\;=\bar{x}\;\pm \beta \sqrt{\sigma^{2}+{\left(\bar{x}-T\right)}^{2}}/2,\;$$

where $\beta \;=\;6\cdot C_{pm}$ and $C_{pm}$ is the process capability ratio which measures the odds of the data lying outside the action limit. β=6 and $C_{pm}=\;1$ are used currently as recommended by TG218.^9^ σ^2^ and $\bar{x}$ are the variance and mean of the measured GPR respectively, and *T* is the target value which is 100% in this case. $\bar{x}$ is also known as the center line and the tolerance limit of the measurement is defined with respect to the center line:

(2) $$T\;L_{I}=\bar{x}\;\pm 2.660\cdot \bar{mR}.\;$$

$\bar{mR}$ is the moving range and is defined as $\frac{1}{n-1}\sum_{i\;=\;2}^{n}\left|x_{i}-x_{i-1}\right|$, where *n* is the total number of measurements and $x_{i}$ represents the *i*‐th measured value. It is important to note that in this context, the upper limits of the action and tolerance limits are bounded by 100%. The tolerance limit for mR is defined as

(3) $$T\;L_{mR}=\;\bar{mR}\pm 3.27\cdot \bar{mR}.$$

The lower tolerance limit of the MR chart is bounded by zero as MR is strictly non‐negative. The chart with the measurement data point and the action and tolerance limit constitute the I‐chart, while the chart with the moving range, mR, and its corresponding tolerance limit is known as MR‐chart. While the utility of I‐chart is well‐understood, the MR‐chart helps in identifying huge variability in inter‐patient measurement results, which could be indicative of an abnormality. When plot together, they are known as the I‐MR chart, which is used for monitoring the measurement value and its variation over time. Readers can refer to the textbook by P. Qiu^40^ for derivations of Equations (1) to (3) and in‐depth explanation of the process capability ratio. The action and tolerance limits are calculated using Equations ((1), (2), (3)) for each of the two sites and three different gamma criteria of 2%/2 mm, 2%/1 mm, and 1%/1 mm. These yield a total of six different tolerance and action limits.

### 2.5 Outlier detection modeling

Using the tolerance and action limits derive for each gamma criterion and site, we develop site and criterion‐specific outlier detection models to detect *outlier* measurement lying beyond the tolerance limits using the 17 plan complexity metrics extracted for each field. Due to extreme class imbalance with insufficient outlier data for training a classification model reliably, an outlier detection model is used. Three different models, namely, one class support vector machine (SVM),^41^ isolation forest,^42^, ^43^ and robust covariance^44^ are compared using the *scikit‐learn v1.1.1* module in Python.^45^ These models are chosen as they are one of the more commonly used outlier detection algorithms^36^ and have found success in heathcare data application.^46^, ^47^ Furthermore, they can be implemented using the python module and therefore this workflow can be replicated easily by physicist in the clinic. One class SVM uses the hyperplanes^48^ or hypersphere^49^ to encompass all the instances with the possibility of margin violation to control sensitivity to noise. Complex decision boundary is possible with the use of non‐linear kernels to map the features to higher dimensional space where simpler decision boundary can be found. Isolation forest identifies outlier by determining shortest average path to that instance after recursive partitioning on random feature and randomly determined feature value. Lastly, robust covariance is the least complex method amongst them, which performs a robust covariance of the data and identifies dataset outside of the elliptical envelop as outlier.

For each site and gamma criterion, the training dataset consists of only the inliers (outliers are excluded from the training dataset), and the validation datasets comprise of both the inliers and outliers. The training datasets are then used to train the three different models with different hyperparameters. The hyperparameters are optimized using the F1‐score,^50^ which is calculated by evaluating the model on the training dataset and the unseen outlier dataset. The available hyperparameters for each model are detailed in the *scikit‐learn documentation* online and the values are chosen to cover the entire possible range of the parameters and to suit the size of the problem in hand (which essentially depends on the dimension of the feature space). A grid search approach is used to determine the set of hyperparameters. The search space for the one class SVM model are ν∈[0.1,0.2,0.3,0.5,0.8], Γ∈[1,2,5,10,15,20,50], and kernel∈[rbf,linear,sigmoid], where ν, Γ, and kernel represent the upper bound for the training error, kernel coefficient and the type of kernel functions respectively. The search space for the isolation forest model are $n_{estimators}\in \left[20,50,80,100\right]$, contamination∈[0.005,0.01,0.02,0.05] and max_features∈[0.5,0.8,1.0], where $n_{estimators}$, contamination and max_features represent the number of bases estimators, the estimated proportion of outliers and maximum proportion of features to be used for training each base estimator, respectively. The search space for the robust covariance model is support_fraction∈[0.1,0.2,0.5,0.8] and contamination∈[0.005,0.01,0.02,0.05], where contamination and support_fraction represent the estimated proportion of outliers and proportion of points to be included in the minimum covariance determinant estimate, respectively. This approach of model optimization will reduce underfitting but will not yield information on whether the model is overfitting as all the inliers are used in the training rather than validation datasets. The performance of the optimal model for each site and gamma criterion are quantified using three metrics—precision, recall and F1‐score. Precision is defined as

(4) $$Precision\;=\frac{TP}{TP+FP}\;,\;$$

recall is defined as

(5) $$Recall\;=\frac{TP}{TP+FN}\;$$

and F1‐score is defined as the harmonic mean of precision and recall:

(6) $$F1-score\;=\frac{2TP}{2TP+FP+FN}\;.$$

TP, FP, and FN are the true positive, false positive, and false negative, respectively.

The top performing outlier detection model (using F1‐score metric) in the respective pelvis and thorax sites are selected for further analyses to determine the decision boundaries and feature importance (also known as permutation importance in this work). Due to difficulty in visualizing decision boundary in a 17‐dimensional space, T‐distributed stochastic neighbor embedding (t‐SNE)^51^ is employed as a non‐linear dimensionality reduction technique to map the data into a two‐dimensional space. Then, the *approximate* decision boundary^52^ is determined by coloring each pixel based on proximity of the predicted class of the dataset. Permutation importance is calculated by scoring the loss in F1‐score by randomly shuffling each feature. A larger F1‐score loss implies a greater importance in the feature in driving correct prediction.

## 3 RESULTS

### 3.1 Patient‐specific quality assurance data and methodology

**FIGURE 1:**
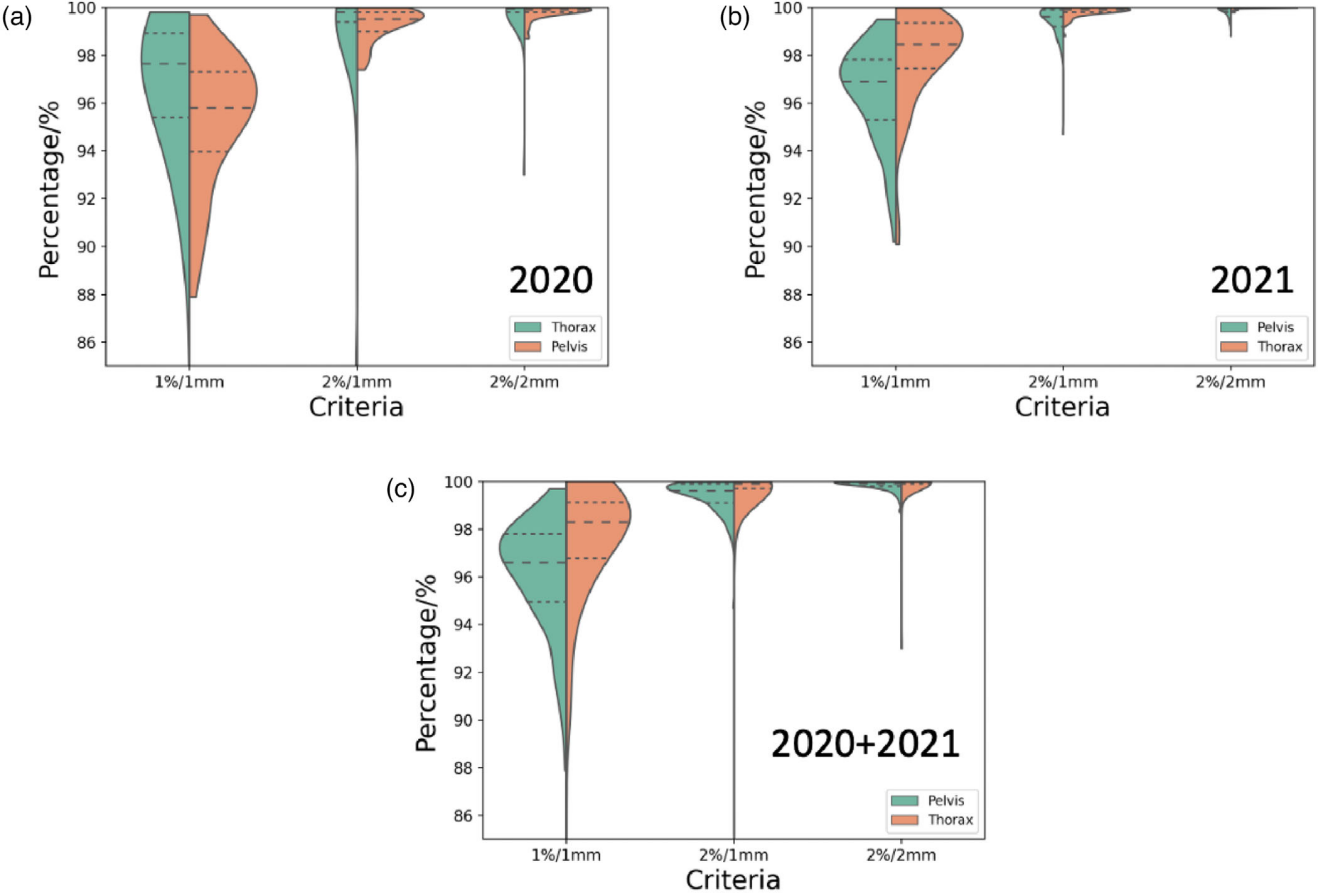
(a–c) show the violin plots of the GPR in year 2020, 2021, and altogether, respectively. The pelvis and thorax GPR are plotted in orange and green, respectively, in the figure. The dashed and dotted lines represent the median and interquartile range, respectively.

The number of measurements doubled in 2021 is not due to a difference in patient loads, but due to a shift in QA measurement technique. Prior to 2021, the plans are measured with a host of different tools including SNC MapCheck 2 or SNC ArcCheck (Sun Nuclear Corporation, Middleton, Wisconsin, USA) whose results are not included in this study. Harmonization of the PSQA measurement technique using PD takes place after January 2021 in our institution. The violin plots of the GPRs with different gamma criteria and in different site are shown in Figure 1.

The median gamma passing rates for all pelvis treatment field evaluated with 2%/2 mm, 2%/1 mm, and 1%/1 mm gamma criteria are 99.9% (interquartile range, IQR = 0.2%), 99.6% (IQR = 0.8%), and 96.6% (IQR = 2.85%), respectively. Similarly, the gamma passing rates for thorax site are 100.0% (IQR = 0.1%), 99.9% (IQR = 0.3%), and 98.3% (IQR = 2.35%), respectively. The thorax treatment fields generally have a higher GPRs than pelvis except for the violin plot for 1%/1 mm in 2020. We suppose this could be due to noisy data from using a more sensitive criterion as the trend disappears when looking at the 2%/1 mm and 2%/2 mm criteria in the same year. The data are clearly not normally distributed especially for 1%/1 mm criterion where heavy tails towards the lower GPR values can be clearly observed.

### 3.2 Statistical process control for determining tolerance and action limit

Using Equations ((1), (2), (3)), the tolerance and action limits for each site and gamma criterion are shown in Table 2.

**TABLE 2:** This table shows the tolerance and action limit calculated from the statistical process control methodology for different sites and GPR criteria.

Criteria	Thorax, *n* = 200		Pelvis, *n* = 308	
	Tolerance limit	Action limit	Tolerance limit	Action limit
2%/2 mm	99.1%	98.1%	99.0%	98.7%
2%/1 mm	95.8%	91.1%	97.0%	96.2%
1%/1 mm	91.7%	86.1%	91.5%	87.2%

**FIGURE 2:**
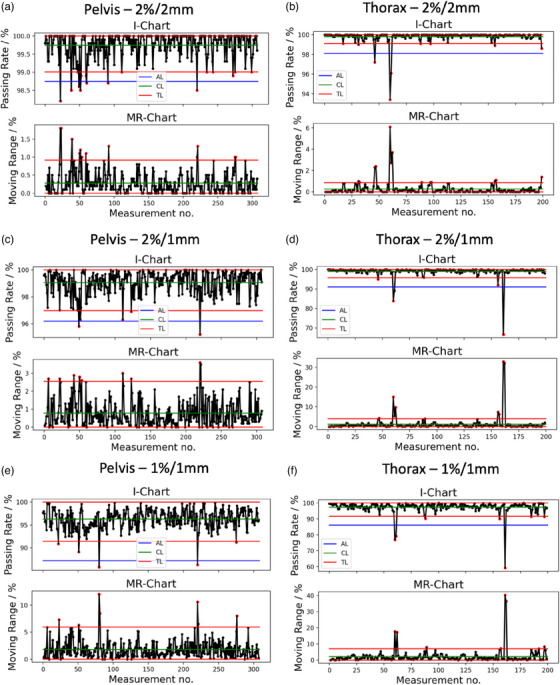
(a,c,e) shows the I‐MR charts for the pelvis site with 2%/2 mm, 2%/1 mm, and 1%/1 mm gamma criteria, respectively. (b,d,f) shows the I‐MR charts for the thorax site with 2%/2 mm, 2%/1 mm, and 1%/1 mm gamma criteria, respectively. The blue, red, and green lines represent the action limit, tolerance limit, and centre line, respectively. The outliers, which are defined as data points lying outside the tolerance limit, are shown as red dots in the figures.

The values of the limits are very similar between the two sites for 2%/2 mm and 1%/1 mm gamma criteria but differ for 2%/1 mm criterion. The tolerance limits are also within the action limits, which means the β value in Equation (1) is sufficient and do not need to be increased. The I‐MR charts for all the PSQA results for the two sites and different gamma criteria are shown in Figure 2.

The center line, tolerance limit and action limits lines are plotted in the figure for reference. The data point with GPR outside the tolerance (also known as outliers in this study) are shown as red dots in the figure. The number of outliers in the pelvis (thorax) treatment field PSQA data with 2%/2 mm, 2%/1 mm, and 1%/1 mm criteria are 16 (9), 7(5), and 5(7), respectively. This constitutes an event rate of 5.19% (3.00%), 2.27% (2.50%), and 1.62% (3.50%). In the thorax site, the outlier data points are generally the same across the different gamma criteria. This is however not true in the pelvis dataset where the outlier events for 2%/2 mm gamma criterion in the pelvis site are greater than when using other criteria. However, all the five pelvis outlier data points in the 1%/1 mm criterion can be found in all the other criteria.

### 3.3 Outlier detection modeling

After optimizing the hyperparameters of the outlier detection models on both the training inlier dataset and the “unseen” outlier datasets, the precision, recall, and F1‐score of the final optimal models are shown in Table 3.

**TABLE 3:** The precision, recall, and F1 score of the optimal model calculated on the training inliers and unseen outliers together for different treatment sites, gamma criteria and models.

Methods	No. of outliers	Precision	Recall	F1
Pelvis, 2%/2 mm	16			
Isolation Forest		0.40	0.25	0.31
Robust Covariance		0.21	0.25	0.23
Support Vector Machine		0.25	1	0.40
Pelvis, 2%/1 mm	7			
Isolation Forest		0.50	0.29	0.36
Robust Covariance		0.17	0.43	0.24
Support Vector Machine		0.54	1	0.70
Pelvis, 1%/1 mm	5			
Isolation Forest		0.33	0.20	0.25
Robust Covariance		0.059	0.2	0.091
Support Vector Machine		0.13	1	0.23
Thorax, 2%/2 mm	9			
Isolation Forest		0.50	0.22	0.31
Robust Covariance		0.23	0.33	0.27
Support Vector Machine		0.56	1	0.72
Thorax, 2%/1 mm	5			
Isolation Forest		0.50	0.20	0.29
Robust Covariance		0.23	0.60	0.33
Support Vector Machine		0.25	1	0.40
Thorax, 1%/1 mm	7			
Isolation Forest		0.50	0.14	0.22
Robust Covariance		0.17	0.29	0.21
Support Vector Machine		0.29	1	0.45

The number of outliers as shown in Figure 2, as the red dots are also indicated using the tolerance limits defined in Table 2.

In general, isolation forest and one class SVM perform better than robust covariance method in term of F1‐score. Furthermore, one‐class SVM achieve a recall of 1.00 in all the site and criteria‐specific model (which means it can detect all the actual outlier events) even though the outlier data are never presented to the model in the training phase. The outlier detection model has the best results in predicting 2%/2 mm GPR of thorax treatment fields. It achieves a precision of 0.56, recall of 1.0 and F1‐score of 0.72. The optimal hyperparameter is ν=0.2, kernel=rbf, and Γ=10. Similarly, the model has the best performance in predicting 2%/1 mm GPR of pelvis treatment fields. It achieves a precision of 0.54, recall of 1.0, and F1‐score of 0.70. The optimal hyperparameter is ν=0.5, kernel=rbf, and Γ=15. Both models have a precision of about 0.50, which means half of the predicted outliers are false positive.

**FIGURE 3:**
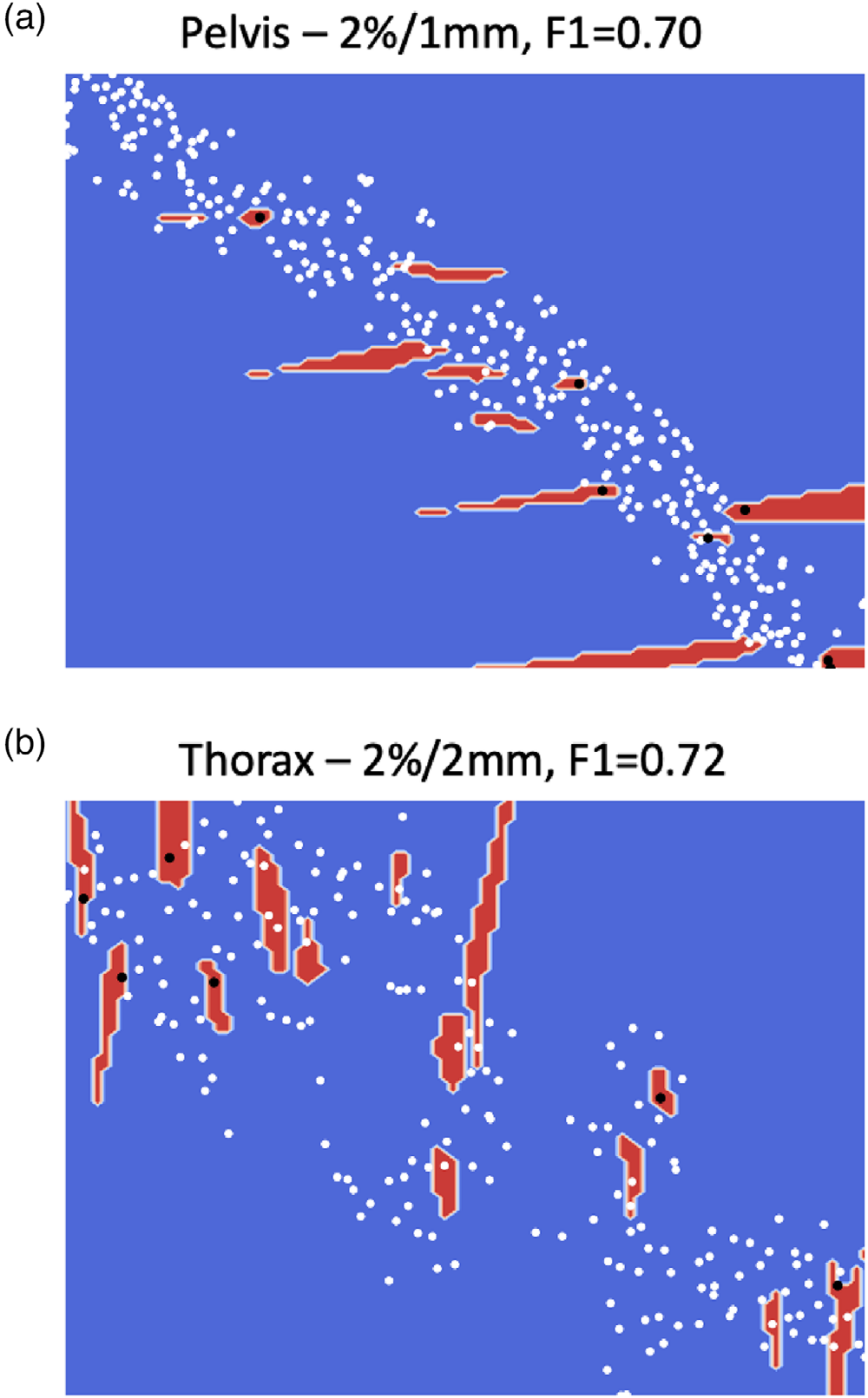
(a,b) show the decision boundary maps of the two best performing models. The best models are stated in the titles of the two figures. The white and black dots represent the actual inlier and outlier, respectively. The red regions show the decision boundaries corresponding to the outliers. Hence, a black dot lying within the red region is a true positive and a white dot lying within the blue region in a true negative.

**FIGURE 4:**
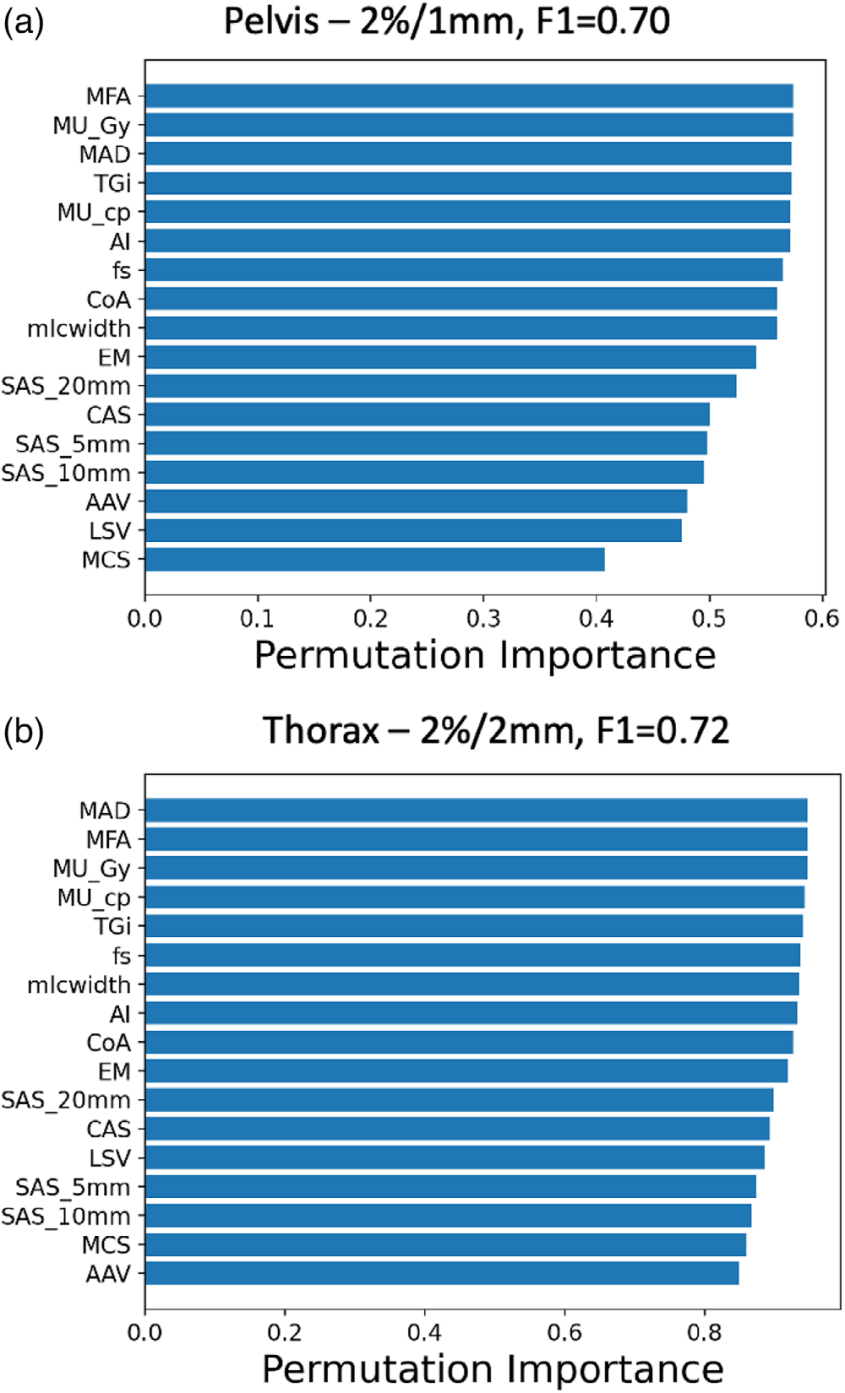
Permutation importance of the two best outlier detection models. (a,b) show the permutation importance of the two best performing models. Three most important features contributing to the correct classifications are MFA, MU_Gy, and MAD, which are similar in both models.

The decision boundaries of these two models are shown in Figure 3. We can see that all the outliers (black dots) and occasional white dots (inliers) lie within the red regions. This agrees with the results in Table 3 where the recall (or sensitivity) of the model is 1.00 and the precision is less than unity. The fragmented nature of the decision boundaries indicates the complexity of the model in calculating the hypersphere in higher dimensional space to encompass the inliers. Lastly, the permutation importance calculated for the two best performing models are shown in Figure 4. Interestingly, despite the different planning protocols for the different treatment sites, the top three features in driving the detection models are similar—MFA, MU_Gy, and MAD. Apart from this observation, one can see that multiple features in each model are contributing almost equally towards the detection model (as can been seen by the almost equal permutation importance score for the first 6 and 10 features in Figure 4a and 4b, respectively), and the model's prediction is not dominated by any single feature.

## 4 DISCUSSION

In this study, we have determined our institutional tolerance and action limits for PSQA with PD for different gamma criteria and for treatment field from two different treatment sites. Our previous PSQA tolerance limit is GPR > 95% with the 3%/2 mm criterion for all treatment sites as advised by TG218. This is a universal tolerance limit, and our clinical experience with this limit show that the limit is reasonable for ArcCheck but is too generous for PD. The new tolerance limits in this study are stricter than the previous one and are more sensitive in detecting anomalous PSQA results. The sample size used in this study is larger than the minimum sample size of 100 recommended by Quesenberry et al.^53^ to set a reliable testing individual control chart. In the thorax site, the dose discrepancies of the outliers are large enough to be discerned using all the tolerance limits set under different three gamma criteria. However, in the pelvis site, the 2%/2 mm criterion and its tolerance limit picks up dose discrepancies that are otherwise undetected by the more stringent criteria. This could be due to a proportion of the dosimetric discrepancy in the overall pelvis data are lying between the 1 and 2 mm distance‐to‐agreement (DTA) margin, such that the 2%/2 mm GPR in the pelvis data has less variation compared to the other criteria. Hence, the tolerance limits are set too high which makes it more sensitive to the dose discrepancy in the outlier data points. A retrospective investigation on the Truebeam daily MPC (machine performance check) and monthly QA data were carried out on the outliers to ensure the slightly lower GPR are not due to calibration or MLC mechanical problems.

Out of the three outlier detection algorithms, robust covariance performs the worst (see Table 3), as it assumes a Gaussian‐shaped decision boundary and thus does not have sufficient model complexity to model intricate boundaries in the data. One class SVM performs the best and achieves a recall of 1.0 across all the different sites and gamma criteria. The recall result is remarkable as no outlier data are presented to the algorithm during training (unsupervised learning) and yet the decision boundary learnt by the algorithm can partition the outliers effectively. Figure 3 shows the inliers and outliers in the two‐dimensional manifolds and that complex decision boundaries are required to correctly identify the outliers. Furthermore, these results show that the plan characteristics quantified by the complexity metrics are predictive of low GPR beyond the tolerance limits. There have been mixed results from studies showing plan complexity metrics being both predictive^15^, ^54^, ^55^ and non‐predictive^56^, ^57^ of PSQA GPR. This could be due to myriads of confounding variables present in this kind of study such as planner's experience, tumor sizes, QA set‐up or calibration errors and so on, which could affect the conclusion significantly. Nonetheless, our study supports the proponent of plan complexity metrics being a predictor of PSQA. Figure 4 show that accuracy metrics (MFA, MAD and MU_Gy are part of the accuracy plan complexity metrics^12^) slightly more than deliverability metrics are driving the predictive model. In essence, the model is looking at the segment open area size (MFA), the degree of off‐axis open segment (MAD) and the degree of MLC modulation (MU_Gy) to make the decision. The best precisions achieved are about 0.50 in both treatment sites, which implies that 50% of the detected outliers will be false alarms. Practically, this means that the manpower and time devoted to investigating or re‐optimising the “predicted failing plan” will be wasted for 50% of the detected outlier. The acceptance of the degree of false alarms depends on individual clinic's workflow and manpower arrangement. With an outlier event rate of 3.0%, the fraction of false alarms out of the total measurements works out to be 3.0% as well which is acceptable in our clinic. With these results, we will be deploying the top two performing models to predict out‐of‐tolerance PSQA results in pelvis and thorax SBRT treatment plans during the planning phase. The planners could refine the plans early if the model predicted a possible violation of tolerance limits.

Majority of the publications treat the predictive modelling of GPR as a regression rather than a classification problem.^14^ With a regression model, a numerical value of GPR will be output and a threshold will be decided a posteriori to decide the passing or failing of the plan. A classification model will require an a priori definition of the threshold and the model will directly output the probability of “passing” the plan. The advantage of the latter approach is the model optimization and evaluation is directly carried out on the outcome of interest, but the disadvantage is the possibility of class imbalance for the selected threshold. At the point of writing the manuscript, there are two reports on the use of classification model for GPR prediction. H. Hirashima et al.^18^ reported a precision and recall of 0.44 and 0.96, respectively, with an event rate of 11.4% using XGBoost model in the validation dataset when using an action limit of 90% with a 3%/2 mm gamma criterion. J. Li et al.^58^ reported a precision and recall of 0.40 and 1.0, respectively, with an event rate of 7.45% using random forest model in the validation dataset when using similar threshold and gamma criterion. Both authors acknowledged the challenge of training a classification model due to the imbalanced training datasets after setting the GPR thresholds. Comparing our model with the two published work, we achieve a similar recall rate and slightly better precision score in the validation cohort despite a lower event rate. It is difficult to draw a conclusion on which model is more superior due to the difference in datasets, but outlier detection model can certainly be an alternative solution to classification model in the light of imbalanced training data which dominates the PSQA datasets.

This study applies the SPC and outlier detection models to PD PSQA methods, but this could easily be extended to other QA devices or other form of QA data. Although TG218 recommended a true composite measurement for PSQA, PD has been reported to have a better detection of MLC shift errors compared to ArcCheck,^25^, ^59^ MapCheck2 (Sun Nuclear Corporation, Middleton, Wisconsin, USA) and MatriXX (IBA, Louvain‐La‐Neuve, Belgium). Hence, PD PFF measurement remains the main QA methods for pelvis and thorax SBRT in our institution.

There are two main limitations in these works. Firstly, it is important to note that the SPC methodology in this work and TG218 assume data normality, which is untrue from Figure 1. Q. Xiao et al.^60^ has developed methods to calculate tolerance limits for a non‐normal distribution data by transforming the distributions. They reported that directly applying the Shewhart control chart without correcting for the non‐normal distribution could result in a higher Type I risk and false positive rate. From a clinical workflow point of view, this could result in unnecessary man hours to investigate an out‐of‐tolerance plans. Despite this, one could argue that the false negative rate remains unchanged which is still acceptable. Secondly, the sample size of outliers determined from SPC is small, and it is ideal to have a hold‐out dataset with a larger number of outliers events to test the generalizability of the model. However, as also pointed by J. Li et al.,^58^ outliers or “failing plans” are uncommon in clinical settings and they encourage multi‐institutional study to collect an adequate amount of low GPR plan for training a reliable model. Furthermore, a greater sample size will empower the study with a greater statistical power for drawing a firmer conclusion when comparing between models. Hence, further data collection and collaboration will be required to establish the future evaluation cohort.

## 5 CONCLUSION

We have determined our institutional tolerance and action limits for our patient‐specific quality assurance with portal dosimetry. With these limits, we have trained a site‐specific outlier detection model that can detect the outliers with a sensitivity of 100% with a false‐positive rate of about 50%. This is the first time outlier detection model is used in the prediction of PSQA GPR, and this provides a more convenient alternative method to train a model in the case of extreme class imbalance in the dataset. This tool will help the planner identify an out‐of‐tolerance plan early so that they can refine the plan further during the planning stage without risking late discovery during measurement.

## AUTHOR CONTRIBUTIONS

Study conception and design: Hong Qi Tan, Kah Seng Lew, Calvin Wei Yang Koh. Data acquisition and analysis: Hong Qi Tan, Yun Ming Wong, Wen Chuan Chong, Clifford Ghee Ann Chua, Ping Lin Yeap. Data interpretation: All authors. Statistical analyses: Hong Qi Tan, Kah Seng Lew. Obtained funding: Hong Qi Tan. Administrative, technical, or material support: Hong Qi Tan. Study supervision: Khong Wei Ang, James Cheow Lei Lee, Sung Yong Park. Drafting of manuscript: Hong Qi Tan. Approval of final manuscript: All authors.

## CONFLICT OF INTEREST STATEMENT

The authors have none to declare.

## Data Availability

Data generated or analyzed during the study are available from the corresponding author by request.
